# Different as night and day: Behavioural and life history responses to varied photoperiods in *Daphnia magna*

**DOI:** 10.1111/mec.15230

**Published:** 2019-09-26

**Authors:** Kurt A. Gust, Alan J. Kennedy, Jennifer G. Laird, Mitchell S. Wilbanks, Natalie D. Barker, Xin Guan, Nicolas L. Melby, Lyle D. Burgoon, Michael E. Kjelland, Todd M. Swannack

**Affiliations:** ^1^ Environmental Laboratory Engineer Research and Development Center US Army Vicksburg MS USA; ^2^ Bennett Aerospace Cary NC USA; ^3^ Present address: Division of Science & Mathematics Mayville State University Mayville ND USA; ^4^ Present address: Conservation, Genetics & Biotech LLC Valley City ND USA

**Keywords:** *Daphnia* behaviour, *Daphnia* life history, male production, photoperiod, phototaxis

## Abstract

Nearly all animal species have utilized photoperiod to cue seasonal behaviours and life history traits. We investigated photoperiod responses in keystone species, *Daphnia magna*, to identify molecular processes underlying ecologically important behaviours and traits using functional transcriptomic analyses. *Daphnia magna* were photoperiod‐entrained immediately posthatch to a standard control photoperiod of 16 light/ 8 dark hours (16L:8D) relative to shorter (4L:20D, 8L:16D, 12L:12L) and longer (20L:4D) day length photoperiods. Short‐day photoperiods induced significantly increased light‐avoidance behaviours relative to controls. Correspondingly, significant differential transcript expression for genes involved in glutamate signalling was observed, a critical signalling pathway in arthropod light‐avoidance behaviour. Additionally, period circadian protein and proteins coding F‐box/LRR‐repeat domains were differentially expressed which are recognized to establish circadian rhythms in arthropods. Indicators of metabolic rate increased in short‐day photoperiods which corresponded with broadscale changes in transcriptional expression across system‐level energy metabolism pathways. The most striking observations included significantly decreased neonate production at the shortest day length photoperiod (4L:20D) and significantly increased male production across short‐day and equinox photoperiods (4L:20D, 8L:16D and 12L:12D). Transcriptional expression consistent with putative mechanisms of male production was observed including photoperiod‐dependent expression of transformer‐2 sex‐determining protein and small nuclear ribonucleoprotein particles (snRNPs) which control splice variant expression for genes like transformer. Finally, increased transcriptional expression of glutamate has also been shown to induce male production in *Daphnia pulex* via photoperiod‐sensitive mechanisms. Overall, photoperiod entrainment affected molecular pathways that underpin critical behavioural and life history traits in *D. magna* providing fundamental insights into biological responses to this primary environmental cue.

## 1 INTRODUCTION

The cladoceran *Daphnia magna* is a keystone ecological model species commonly utilized for freshwater ecology research and standardized toxicological testing. *Daphnia magna* integrates environmental sensing and response mechanisms to enable behavioural and life history modifications commensurate with environmental challenges and seasonal changes. Changes in behaviour and life history strategy of *Daphnia* species are often studied in context with multiple, combinational cues, such as temperature, photoperiod, resource availability and population density (Alekseev & Lampert, 2006; Altshuler et al., 2011; Gust et al., 2016; Heugens, Hendriks, Dekker, Van Straalen, & Admiraal, 2001; Heugens et al., 2006; Jiang et al., 2014; Korpelainen, 1986). In a study of interactive effects between temperature and photoperiod (Armitage and Landau, 1982), it was concluded that (a) interactive effects occurred impacting lifespan, size, reproductive output and broods; (b) temperature had independent effects; and (c) photoperiod alone was unlikely to have independent effects. The present study was conducted to begin untangling responses to environmental cues by investigating the most basal and consistently rhythmic environmental cue, photoperiod. Across evolutionary time, nearly all animal species have harnessed photoperiod to initiate various physiological and developmental processes that ultimately influence behaviour and life history traits (Bradshaw & Holzapfel, 2007). Our investigations assessed the influence of photoperiod on the immediate behavioural responses to external stimuli and lifelong changes to *D. magna* life history strategy. The molecular mechanisms underlying behavioural and life history responses to photoperiod are not well understood in *D. magna*; therefore, a global transcriptomic expression investigation was conducted to identify functional responses involved in organism‐level outcomes.

*Daphnia* species exhibit negative phototaxis, a behaviour that is generally hypothesized to facilitate predator avoidance (Dodson, Tollrian, & Lampert, 1997; Van Gool & Ringelberg, 1995). Deviations from negative phototaxis have been observed in *Daphnia* species (Michels, Leynen, Cousyn, Meester, & Ollevier, 1999; Ringelberg, 1964) that may be attributed to environmental modifiers, such as light wavelength (Storz & Paul, 1998) and food availability (Jiang et al., 2014; Neary, Cash, & Mccauley, 1994; van Gool & Ringelberg, 1995). Despite the broad evidence that photoperiod can affect animal behaviour, the influence of photoperiod on phototaxis remained to be investigated in *D. magna* prior to the present study. Further, the direct influence of photoperiod on critical life history parameters in *D. magna* also remained unclear. For example, *D. magna* normally reproduce via asexual clonal parthenogenesis, however, combinations of environmental factors such as temperature, short‐day length photoperiod, crowding and food availability can stimulate male production enabling genetic recombination in response to a changing environment (Carvalho & Hughes, 1983; Hobæk & Larsson, 1990; Kleiven, Larsson, & Hobæk, 1992). Short‐day photoperiod has been reported to play a critical role in triggering male production in *Daphnia pulex* (Toyota, Gavin, Miyagawa, Viant, & Iguchi, 2016; Toyota, Miyakawa, Yamaguchi, et al., 2015) and recently also in *D. magna* when in conjunction with increasing temperature. Ultimately, varying investments to sexual reproduction are known to have important ecological and evolutionary consequences (Tessier & Cáceres, 2004). These observations compelled us to investigate the system‐level responses to photoperiod in order to evaluate its effect(s) across behavioural, metabolic, survival, growth, reproduction and male production responses in *D. magna* under controlled conditions.

We conducted experiments to test the null hypothesis that photoperiod alone had no effect on specific behavioural and life history outcomes in *D. magna*. The experiments were conducted in custom‐constructed photoperiod chambers where <24‐hr‐old *D. magna* were reared in five different photoperiods ranging from short‐day, equinox and long‐day states. Phototaxis responses were observed ex situ using digital tracking of velocity and duration spent in light/dark zones (noldus daniovision ^®^ software), and carbohydrate metabolic rate was quantified using IQ Toxicity Test™ (Aqua Survey, Inc.) to estimate overall metabolic rate, activity and health. These short‐term behaviours/strategies were compared against long‐term life history parameters including survival, growth, reproduction and reproductive strategy (female vs. male neonate production). Microarray‐based global gene expression assays were conducted to examine the functional genomic processes underlying both the behavioural and life history responses of *D. magna* to photoperiod. Overall, the approach yielded unique system‐level insights into the influence of photoperiod from the molecular to lifetime responses in *D. magna*.

## 2 MATERIALS AND METHODS

### 2.1 Experimental approach and rationale

The experimental approach focused on investigating the effects of photoperiod on key life history traits, metabolic rate, phototaxis responses and global transcriptomic expression in *Daphnia magna* to characterize foundational responses to light. Separate groups of *D. magna* were simultaneously exposed to five different photoperiods representing varying seasonal day lengths; this included a comparative control of 16 hr of light and 8 hr of dark (16L:8D) that is the standard for acute (OECD No. 20, 1984; USEPA, 2002; USEPA, 1996a) and chronic (ASTM E1193‐97, 2012; OECD No. 211, 2012; USEPA, 1996b) ecotoxicological methods, versus an array of alternative photoperiods including extreme short‐day length (4L:20D), short‐day length (8L:16D), equinox (12L:12L) and extreme long‐day length (20L:4D). Since changes in life history strategy are likely to involve changes in energy utilization (Ananthasubramaniam et al., 2015), an indicator of overall metabolic rate was also investigated. Provided the inherently variable nature of life history and behavioural responses to stimuli, we repeated the photoperiod exposures in two temporally separated experiments on 17 October 2014 and 11 March 2015, hereafter referred to as experimental trial 1 and experimental trial 2. This was done to assess the reproducibility of all measures and endpoints from one experiment to the next, and assess if the two data sets could be combined for examination as one large data set.

### 2.2 Experimental animals

*Daphnia magna* neonates (<24 hr old) obtained from in‐house cultures reared for several years in a 16L:8D light cycle were used in the photoperiod experiments. Organisms were originally purchased from a commercial source in May 2009 (Aquatic Biosystems; EPA Ohio, AROF2, Lot No. 070092DM), and in‐house cultures were started using one individual to ensure testing utilized a single genotype. All culturing and test methods used hard reconstituted water (HRW), formulated according to USEPA (2002). Test organisms in cultures and experiments were held at 25 ± 1°C and provided a daily ration of 2 × 10^5^ algae cells/ml (*Raphidocelis subcapitata*, formerly *Selenastrum capricornutum*) and yeast‐cerophyl‐trout chow (YCT; Aquatic Biosystems) as prescribed by guidance (ASTM, 2012; USEPA, 2002). The algae used as food were cold stored (4°C) in the dark to inhibit growth and were directly spiked from cold storage into the experimental chambers that contained *D. magna*. Proliferation of the algae in the experimental chambers was not quantified, but proliferation was unlikely given rapid feeding by *Daphnia* immediately after spiking, tri‐weekly water renewals flushing out old algae, static water conditions, test media devoid of nutrients and low relative light intensity in experimental chambers.

### 2.3 General exposure methods

Specialized exposure chambers resembling ant farms were constructed from clear acrylic (JR's Custom Acrylics). The chamber dimensions were 35 × 17 × 2 cm (Figure S1) and contained approximately 1,190 ml HRW (34.5 cm water height); each was initially loaded with 20 *D. magna neonates*. To isolate the exposures from ambient laboratory lighting, sets of 3 replicate exposure chambers were placed within five separate wooden boxes (36 × 70 × 70 cm; Figures S1 and S2) where each photoperiod treatment was administered. Each photoperiod box had a black interior and was equipped with a wide‐spectrum fluorescent lamp (Exo Terra PT2151, Rolf C. Hagen Corp.) providing overhead lighting that was uniform across all three exposure chambers. Photoperiods were separately regulated by digital light timers (Woods Digital Indoor Timer #50008, Coleman Cable, Inc.). The light penetration within the test chambers ranged from 730 to 6,750 lux, depending on depth (Figure S3).

Duplicate photoperiod experimental trials 1 and 2 were conducted for 21 days, adapted from the standard ASTM method E1193‐97 (ASTM, 2012) duration, at 25 ± 1°C within Darwin Environmental Chambers (Darwin). Complete (100%) water renewals using HRW were performed three times a week (Monday, Wednesday, Friday); viable neonates were enumerated and removed from the experimental chambers at each water exchange. Thus, only the original individuals loaded into the chambers remained for the duration of the experiment. Water quality monitoring was performed, the values of which remained within test method specifications (EPA‐821‐R‐02‐012), with the following ranges: temperature (25 ± 2°C), pH (7.8–8.3), dissolved oxygen (>4 mg/L), specific conductivity (290 ± 20% µS/cm) and ammonia‐N (<2 mg/L).

### 2.4 Life history endpoints

Survival and the number of neonates produced were assessed three times per week, prior to each water change. Female and male neonates were enumerated by removing all neonates from exposure chambers, growing neonates in 2‐L plastic beakers at the prescribed feeding ration for 5–7 days and identifying the sex under a dissecting microscope according to available literature and keys (Ebert, 2005; Olmstead & Leblanc, 2006). Carapace length was determined at test termination by capturing calibrated *D. magna* images from at least five individuals per replicate using a digital camera (Leica Microsystems Ltd., DFC425) attached to a dissection microscope (S6 E, Leica Microsystems Ltd.). Length was measured from the eyespot to the base of the anal spine using image‐pro plus software version 7 (Media Cybernetics, Inc.).

### 2.5 Sugar metabolism as an estimate of metabolic rate

Fluorescence bioenergetics assays (IQ‐Tox™ Assays, Aqua Survey, Inc.) were used to investigate the response to the light stimulus providing information on metabolic rates (Hayes, Douglas, & Fisher, 1996). This test kit employed a fluorometric biomarker methylumbelliferyl galactoside. The concept behind the IQ‐Tox™ Assay is that healthier *D. magna* will take up and metabolize more biomarker and fluoresce with more intensity under UV light, while less healthy individuals will metabolize less biomarker and therefore fluoresce less. At termination of the experiments, 21‐day‐old *D. magna* were added to IQ exposure chambers, and each cell was filled with 10 ml of test water and three drops (≈60 µl) of the IQ substrate (a fluorogenically tagged sugar suspension). After 15 min, *D. magna* were removed and transferred onto the lid of a 24‐well microplate (Product #3524, Corning Inc.) for fluorescence imaging, and excess water was removed. Fluorescence imaging was conducted using a ProteinSimple Red™ imaging system (ProteinSimple [formerly Cell Biosciences]). The resulting images were analysed with image‐pro plus software to quantify fluorescence intensity and density, which was used in the statistical analysis. The data generated herein used the fluorescence intensity as an indicator of sugar metabolism rate of the fluorogenically tagged sugar, thus providing a reliable estimate of *D. magna* metabolic rate to compare among the various photoperiod treatments. Data presented herein were generated from experiment 1, using 8–11 *D. magna* per treatment.

### 2.6 Phototaxis

Phototaxis was assessed for each of the experimental photoperiods at week 1 and week 3. Short‐term ex situ digital tracking of swimming behaviour was conducted using a real‐time digital tracking system (Noldus DanioVision^®^). Horizontal movements in light and dark conditions were tracked to remove gravity as a confounding variable. The DanioVision apparatus projected full spectrum light (measured to be 3,713 lux) from below the experimental chamber and allowed digital test organism tracking to be performed in the dark conditions by contrast to background using infrared light. Experiments were conducted in custom acrylic raceways (2.0 × 8.6 × 12.8 cm) consisting of eight separate 1.0 × 12.8 cm arenas (Figures S1 and S4). Window tinting (Gila Window Film ‐ 20% visible light transmission, Eastman Performance Films, LLC) was placed on the bottom of the raceway so that half of each of the eight arenas was covered, thus providing *D. magna* a choice between light or dark conditions. Eight individuals were randomly selected from each replicate exposure chamber (total of 8 × 3 replicates = 24 individuals per photoperiod), and initially four were placed in the dark (tinted) zone, and four were placed in the light zone. A 5‐min acclimation period was allowed, followed by 10‐min trials during which *D. magna* movement was digitally tracked, and data were recorded at seven samples per second. At the conclusion of the 1‐week phototaxis trial, the *D. magna* that were examined were carefully returned to the primary experimental chambers. The measurement endpoints assessed included time (s) spent in each light/dark zones and velocity (mm^2^/s).

### 2.7 Microarray experiments

The effects of photoperiod on global transcript expression in *D. magna* were investigated using microarray assays. An Agilent Technologies (Agilent Technologies) single colour custom 8 × 15K microarray format previously developed by Stanley et al. (2013) which was subsequently reannotated in 2015 was used for all investigations. The reannotated microarray design used in the present study is available at the Gene Expression Omnibus (GEO, http://www.ncbi.nlm.nih.gov/) under Accession no GPL16579. The experimental design for the microarray experiments utilized animals collected directly from the photoperiod exposures at the termination of the 21‐day assays and within 1 hr after initiation of the light phase of each photoperiod, for both experimental trials 1 and 2. This sampling approach ultimately included developing eggs/neonates within brood pouches; however, inclusion of such developmental processes is supportive of evaluating photoperiod influences on the molecular contributions to reproductive life history outcomes. Within each exposure replicate, two population subsamples consisting of single, unique *D. magna* were used for hybridization to microarrays. These population subsamples were selected at random (using a random number generator) from 2 to 6 *D. magna* sampled for the genomic investigation that met the RNA quality criteria described in the following paragraph. In summary, 2 duplicate photoperiod experimental trials were conducted where each included 5 photoperiods × 3 exposure replicates × 2 population subsamples for a total of 30 total microarray hybridizations per experiment and thus 60 microarray hybridizations overall. A completely randomized design was utilized for microarray hybridizations where the total set of 60 experimental samples were assigned to microarrays at random (using a random number generator).

#### 2.7.1 RNA extraction and microarray hybridizations

Frozen samples were homogenized using a mortar and pellet pestle (Kimble Kontes). Total RNA isolation was conducted using the Qiagen RNeasy Mini Kit (Qiagen) following manufacturer's recommendations. RNA quantity was estimated using a NanoDrop ND‐1000 Spectrophotometer (NanoDrop technologies), and final quantity and quality were assessed using an Agilent 2100 Bioanalyzer (Agilent Technologies). Only samples with a 28s/18s ratio ≥2.0 and RNA integrity number (RIN) ≥7.0 were used for downstream applications. The Agilent Low Input Quick Amp Labeling Kit (one colour) and hybridization protocol (Agilent Technologies) were utilized for microarray hybridizations following manufacturer's recommendations using 80 ng of total RNA as starting material from each biological sample.

#### 2.7.2 Microarray analysis

An Agilent Technologies, High‐Resolution Microarray Scanner (Model G2505C, Agilent Technologies) was used to scan microarray images at 2‐μm resolution. Data were extracted from microarray images using agilent feature extraction software, version 9.5.1 (Agilent Technologies). Microarray data were normalized to the 75th percentile within each array followed by median scaling among all exposures using genespring Software version GX12.5 (Agilent Technologies). Methods for the statistical analysis of microarray data are provided in Section 2.8.

#### 2.7.3 Microarray reannotation

The assembled sequence information used to develop all probes on the *D. magna* microarray was reannotated in December 2016 and used for functional interpretations of the microarray results. Reannotation was conducted using Blast searches against the NCBI nt database filtered for only those GenBank entries in the *Daphnia* genus, where the best match was kept. The reannotation provided greatly expanded, expressed sequence tag matches which were predominated by matches to *D. magna* and *Daphnia pulex* putative protein coding sequences. Unfortunately, as of March 2019, these putative protein sequences are not supported by protein orthologs to enable functional enrichment analysis (i.e., pathway or gene ontology). Therefore, the original annotations were used for the functional analysis.

#### 2.7.4 Functional annotation

The database for annotation, visualization and integrated discovery (DAVID, version 6.8, Huang, Sherman, & Lempicki, 2009) was used to derive associated Kyoto Encyclopedia for Genes and Genomes (KEGG) annotations for gene transcripts that had significant differential expression (see Section 2.8) in response to the photoperiod treatments. To enable the broadest functional investigation of the transcriptomic results, the union of the significant differentially expressed transcripts among experiments 1 and 2 were used as a combined result set to establish functional annotations. For clarity, the union = all differentially expressed transcripts including those identified in experiments 1, 2 or both experiments. The KEGG pathway annotations were compiled, and hierarchical KEGG ontology (KO) associations were developed. The KO associations were used to organize the transcript expression results into key categories for use in assessing the effects of photoperiod treatments on molecular pathway‐level responses. Transcriptional expression was visualized within KO categories, and hierarchical clustering of samples was established using multi‐experiment viewer Software (MeV version 4.9, Open Source Artistic License 2.0).

#### 2.7.5 Text mining of new protein annotations

To add value to the new protein annotations for *D. magna*, text mining was conducted for all transcripts having significant differential expression in response to the photoperiod treatment using a keyword bank (Table S2) developed by the authors of the present study to match key gene functions connected to the observed phenotypes. Specifically, the keyword bank was developed to mine terms related to circadian rhythm, sex determination, moulting/cuticle processes, egg production and visual cues related to photoperiod or light stimulus to identify potential functional responses to the photoperiod treatments. Text matching was applied to the annotation list for the complete set of differentially expressed transcripts to produce the text mining results.

### 2.8 Statistical analysis

Both life history data (survival, reproduction, size) and phototaxic swimming data were statistically analysed using sigmastat Software (V3.5, SPSS). The assumptions of normality (Kolmogorov–Smirnov test) and equality of variance (Levene's test) were tested. All life history data met the assumptions of normality and equality of variance. Swimming data that did not meet normality or variance assumptions were rank transformed. The Holm–Sidak method was used for pairwise multiple comparisons to determine differences between treatments, which were determined at the α = 0.05. One‐way ANOVAs compared photoperiod to life history data, and two‐way ANOVA compared multiple factors (experimental trial × photoperiod). When the experimental trial (i.e., experimental trials 1 and 2 which were conducted separately in time, *n* = 3) was determined to be a nonsignificant factor, life history data were combined (*n* = 6). For photoperiod versus behavioural swim data analysis, one‐way ANOVAs were performed within each experiment, while two‐way ANOVAs compared two factors (photoperiod acclimation × duration in light/dark zones) and three‐way ANOVA compared three factors (experimental trial × photoperiod acclimation × duration in light/dark zones or organism age × photoperiod acclimation × duration in light/dark zones). When the experimental trial was determined to be a nonsignificant factor, swim behaviour data from replicates 1–3 and 4–6 were also combined (*n* = 6).

For the investigation of microarray data, two statistical approaches were conducted, one including data from both experiments 1 and 2, while the other investigated the data from experiments 1 and 2, separately. For the combined‐experiment analysis, a three‐way ANOVA (*p* = .01) was conducted to investigate three variables: (a) the primary variable of interest, photoperiod; (b) variance from population subsampling; and (c) variance among experiments 1 and 2. For the individual‐experiment analyses (investigating experiments 1 and 2 separately), two‐way ANOVAs (*p* = .01) were conducted to investigate the first two variables described above: (a) photoperiod and (b) variance from subpopulation sampling. In all of the analyses described above, effects of the primary variables and all interactions among variables were evaluated. As a note, in addition to the tests described above, ANOVA tests (*p* = .05) including Benjamini–Hochberg multiple‐test corrections were conducted for each investigation which yielded very few differentially expressed transcripts. Therefore, the results described here‐forward represent noncorrected tests with the low *p*‐values (*p* = .01) to balance resolution (number of differentially expressed transcripts) against false‐positive discovery. In supplement to the ANOVAs described above, post hoc pairwise tests consisting of moderated *t* tests (*p* = .05) including ≥1.5 fold change cut‐off were conducted to identify transcripts differentially expressed within each experimental photoperiod (4L:20D, 8L:16D, 12L:12D and 20L:4D) relative to the control (16L:8D). All microarray analyses described above were conducted using GeneSpring GX12.5 (Agilent Technologies). As a final note, the heatmaps presented in the Results include variance associated with two population subsamples; therefore, the averaged fold change for the control photoperiod (16L:8D) may deviate slightly from one (zero once log_2_ transformed).

## 3 RESULTS

### 3.1 Life history endpoints (survival, growth, reproduction, male production)

**Figure 1:**
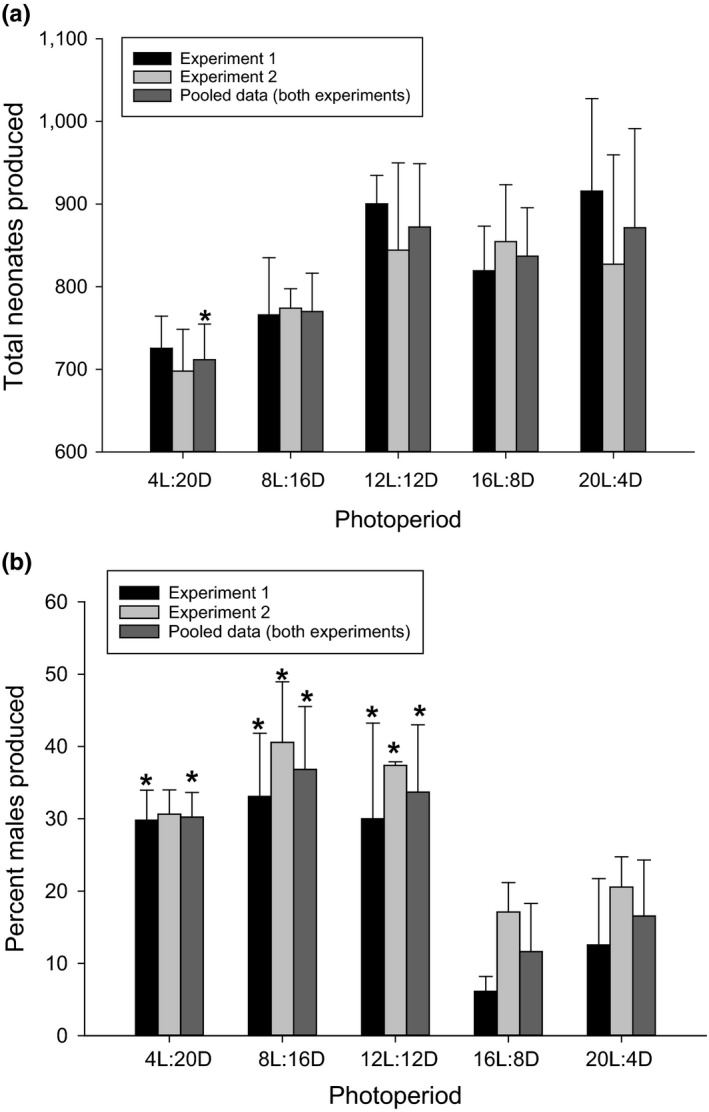
Cumulative reproductive output expressed as (a) average total neonates by experiment and pooled average total neonates from both experiments and (b) per cent male neonates across the five experimental photoperiods. Error bars represent standard deviations, and asterisks denote a statistically significant difference (*p* = .05) relative to the control (16L:8D photoperiod)

The life history endpoint results were summarized for each of the two individual experimental trials (*n* = 3) and as combined data (*n* = 6) that included both experimental trials. Combined survival (*n* = 6) was relatively high (80%–92%). Carapace length (Figure S5) from both experiments ranged from 4.7 to 6.0 mm where in experimental trial 1, *Daphnia magna* were slightly but significantly larger than in experimental trial 2. Generally, there were no significant differences determined by the Holm–Sidak method (*p* > .05) in carapace length relative to the 16L:8D control, with the exception of significantly smaller *D. magna* in the 4L:20D photoperiod in experimental trial 1 (Figure S5). Neonate production among experimental trials 1 and 2 was not significantly different (*p* = .37); thus, the combined data sets were analysed (*n* = 6) where photoperiod caused a slight, but significant decrease (*p* < .01) in total neonate production in the 4L:20D photoperiod relative to the 16L:8D control photoperiod (Figure 1a). Perhaps the most interesting effect of short‐day and equinox photoperiods on life history traits was a significant increase in male neonate production relative to the long‐day (16L:8D) control photoperiod (Figure 1b) which was observed in each experimental trial (*n* = 3) and within the combined data set (*p* < .01, *n* = 6).

### 3.2 Sugar metabolism as an estimate of metabolic rate

**Figure 2:**
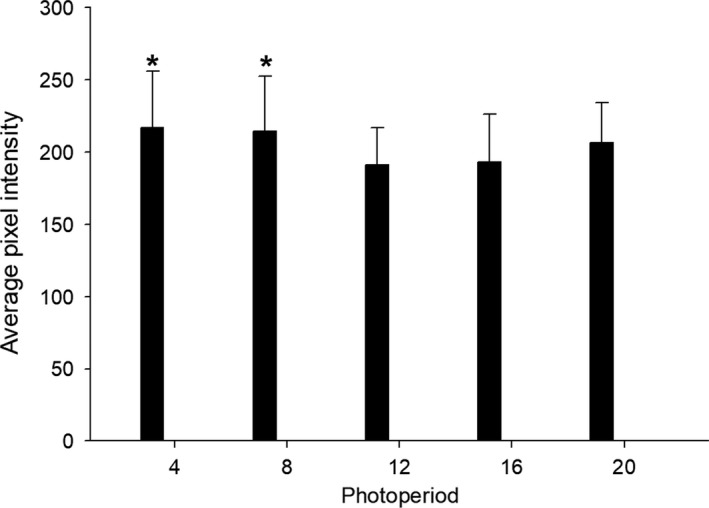
Effect of photoperiod on metabolic rate in *Daphnia magna* (from experimental trial 1, *n* = 8–11), as estimated by metabolism of fluorescently labelled sugar molecules and quantified as pixel intensity from whole‐animal images. Error bars represent standard deviations, and asterisks denote a statistically significant differences (*p* = .05) relative to the control photoperiod (16L:8D)

Analysis of the data from experimental trial 1 (*n* = 8–11) identified a statistically significant increase in pixel intensity (fluorescence output) using the Holm–Sidak method in the exposed *D. magna* in the short‐day length photoperiods, 4L:20D and 8L:16D, relative to the control 16L:8D photoperiod (Figure 2) which is indicative of increased sugar metabolism, suggesting an increased overall metabolic rate.

### 3.3 Phototaxis – digital tracking of swimming behaviour (ex situ)

**Figure 3:**
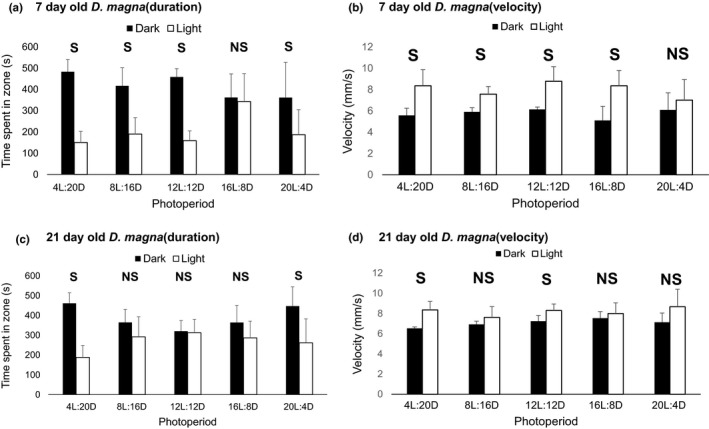
Phototaxis investigation summarizing *Daphnia magna* behavioural data in the phototaxis chamber for 7‐day‐olds (panels a and b) and 21‐day‐olds (panels c and d). The duration of time spent in light versus dark zones is summarized in panels (a) and (c), while swimming velocity is summarized in panels (b) and (d). These results represent the combined data for the two experimental trials (*n* = 6). Black bars indicate the dark zone, while white bars indicate the light zone. Error bars represent standard deviations. The “S” and “NS” designations denote significant and nonsignificant differences (*p* = .05), respectively, in time spent in light versus dark zones within each photoperiod

Photoperiod entrainment showed significant effects on light avoidance in *D. magna* in both experimental trials 1 and 2 (Figure S6), and in the combined data set (Figure 3) as indicated by increased photonegative responses relative to the 16L:8D control. Organism age modulated this photonegative response across the photoperiod exposures where the younger (7 days old) *D. magna* displayed greater light sensitivity relative to 21‐day‐olds, by preferentially residing in the dark for all experimental photoperiods (Figure 3a,c). Correspondingly, results of 2‐way ANOVA confirmed age as a significant factor, along with photoperiod, where these factors also displayed a significant interaction showing complex phototaxis responses to photoperiod that were nonlinearly related to age. Evaluation of *D. magna* swim velocity also indicated a negative phototaxis response where swim speeds were significantly higher in the light versus dark zones in both individual trials (Figure S7) and for the combined data sets (Figure 3b,d). Again, this response was modulated by animal age where a greater number of significant differences in velocity among light and dark zones were observed in the younger 7‐day‐old *D. magna* relative to 21‐day‐olds. Photoperiod significantly affected swim speed, which again was significantly modulated by animal age where the independent variables (photoperiod and age) displayed a significant interaction. Overall, the extreme short‐day photoperiod (4L:20D) showed the most consistently significant photonegative effect on the swim velocity response across animal ages (Figure 3).

### 3.4 Transcriptomic expression

#### 3.4.1 Microarray analysis overview

Combining transcriptomic data for experimental trials 1 and 2 in a 3‐way ANOVA indicated expression differences were dominated by the variance among experimental trials which was also confirmed by PCA (Figure S8). Given these results, the effects of photoperiod for each experimental trial were evaluated separately. The photoperiod treatment caused significant, differential expression of transcripts in the separate experimental trials 1 and 2. Results of the two‐way ANOVAs (*p* = .01) for experimental trial 1 identified 456, 61 and 219 differentially expressed transcripts in response to photoperiod, within‐population subsampling and photoperiod × within‐population subsampling treatments, respectively. Experimental trial 2 yielded 1,468, 275 and 385 differentially expressed transcripts for the same respective treatments. Given multiple tests (14,398 microarray targets), 143 false‐positive tests were expected by chance; thus, the variance associated with “within‐population subsampling” and “photoperiod × within‐population subsampling” was near background. Correspondingly, “within‐population” subsamples tended to cluster together within PCA plots, while photoperiod‐specific clustering tended to show separation from the 16L:8D control (Figure S8). In experimental trial 1, pairwise comparisons against the control photoperiod (16L:8D) yielded 63, 116, 97 and 30 differentially expressed targets in the 4L:20D, 8L:16D, 12L:12D and 20L:4D photoperiods, respectively, and 466, 332, 530 and 70 differentially expressed targets, respectively, in experimental trial 2. The union of the pairwise comparisons results from these 2 experimental trials (Table S1) were used to establish functional annotations for photoperiod responses. Given the nature of the statistical tests conducted, some targets included in the analysis represent random false positives. However, in the following processing of the experimental data, gene function was matched to phenotypic observations of *D. magna* in our experiments which serves to minimize incorporation of random genes (random functions) into the results interpretation. All microarray data/ results are publicly available at the Gene Expression Omnibus (GEO, http://www.ncbi.nlm.nih.gov/geo/info/linking.html) under series accession GSE117244.

#### 3.4.2 Functional annotation of differentially expressed transcripts

The functional annotation results indicated that the majority of differentially expressed transcripts had associations with the primary KO categories: (a) metabolism, (b) genetic information processing and (c) environmental information processing (Figure S9). Over half of the differentially expressed transcripts had KO associations related to metabolism where genetic information processing represented 12%–28% of transcript associates across photoperiods. Although the directional fold change of transcriptional expression was mixed for metabolism and environmental information KO categories, expression for transcripts within the genetic information processing KO category was predominantly decreased relative the 16L:8D control photoperiod (Figure S10). Investigation of transcriptional expression within specific KEGG pathways again showed mixed expression for the metabolism and environmental signalling KOs, where expression tended to be decreased for a variety pathways related to genetic information processing including spliceosome, ribosomal biogenesis, RNA transport, proteasome, RNA degradation and DNA replication pathways (Figure S10). Hierarchical clustering within these primary KOs indicated that transcript expression tended to be more similar within the short‐day length and equinox photoperiods (4L:20D, 8L:16D and 12L:12D) compared to long‐day length photoperiods (16L:8D and 20L:4D, Figure S10).

#### 3.4.3 Text mining of new annotations

Text mining for gene transcripts significantly affected in the photoperiod treatments provided putative matches to the whole‐organism observations (Table 1). Transcript expression for genes matched to circadian rhythm, sex determination and visual cues tended to have decreased expression in short‐day length and equinox photoperiods compared to the 16L:8D control and the 20L:4D photoperiods. A similar response to short‐day length was observed for transcripts matched putatively to “egg” processes; however, the transcript “vitellogenin fused with superoxide dismutase” had increased expression in the short‐day length photoperiods (Table 1). Cuticle and chitin processes are recognized to be important in *Daphnia* species moulting and growth (Giraudo, Douville, Cottin, & Houde, 2017). Gene transcripts involved in cuticle and chitin processes had increased or decreased expression relative to the control 16L:8D photoperiod including increased relative expression of transcripts coding chitin deacetylase 3 and decreased expression of endocuticle structural glycoprotein SgAdb‐2 in the short‐day length photoperiod treatments (Table 1). Functional associations of these transcriptional observations to phenotypes are provided in the discussion.

**Table 1:** Transcript expression for genes putatively identified to be involved in specific photoperiod responses

Exp. trial	Categories Protein accession	*p*‐value Photoperiod	*E*‐value Annotation match	Transcript name	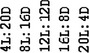
				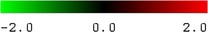	
Circadian rhythm					
Exp1	DM12109	.00499	5.1E‐20	F‐box/LRR‐repeat protein 12	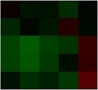
Exp2	KZS07878	.00268	5.1E‐20	F‐box/LRR‐repeat protein 12	
Exp2	JAN38449	.00003	5.3E‐06	Period circadian protein	
Exp2	JAN78534	.00000	6.0E‐132	Period circadian protein	
Exp2	KZS06605	.00042	2.6E‐46	Period circadian protein	
Sex determination					
Exp1	DM04300	.00853	7.1E‐107	Testis development protein nyd‐sp29	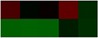
Exp2	JAN91947	.00662	8.6E‐20	Transformer‐2 sex‐determining protein, partial	
Cuticle & chitin					
Exp1	DM05241	.00148	1.1E‐46	Collagen alpha‐1(XVIII) chain	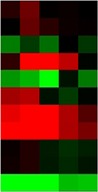
Exp1	DM13721	.00021	5.2E‐32	Brain chitinase and chia	
Exp2	JAN52418	.00248	9.0E‐15	Chitinase domain‐containing protein	
Exp2	JAN66077	.00829	3.8E‐13	Cuticular protein analogous to peritrophins 1‐G	
Exp2	JAN51902	.00255	9.1E‐18	Endocuticle structural glycoprotein SgAbd‐2	
Exp1	DM03120	.00724	5.0E‐85	Putative chitin deacetylase	
Exp2	JAN57634	.00310	2.6E‐102	Putative chitin deacetylase 3 precursor, partial	
Exp2	JAN35264	.00140	1.8E‐08	Putative cuticle protein	
Exp2	JAN61066	.00030	5.6E‐76	Putative cuticle protein	
Exp2	JAN61066	.00044	1.5E‐48	Putative cuticle protein	
Exp2	JAN64809	.00936	4.2E‐46	Putative cuticle protein	
Egg					
Exp2	KZS06734	.00555	2.8E‐25	Female sterile (2) ltoPP43	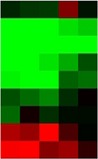
Exp2	JAN29198	.00120	3.0E‐105	Chorion peroxidase precursor	
Exp2	JAN47987	.00203	4.5E‐09	Chorion peroxidase precursor‐like protein, partial	
Exp2	JAN47987	.00984	6.3E‐116	Chorion peroxidase precursor‐like protein, partial	
Exp2	JAN90603	.00038	4.9E‐35	Putative vitelline membrane outer layer protein	
Exp2	KZS10607	.00601	4.1E‐27	Vitelline membrane outer layer protein 1	
Exp1	DM00375	.00200	1.2E‐44	Vitellogenin fused with superoxide dismutase	
Exp2	BAE94324	.00720	1.6E‐108	Vitellogenin fused with superoxide dismutase	
Exp1	DM03056	.00540	2.5E‐27	Putative Notch, partial	
Visual cues					
Exp1	DM01796	.00211	3.7E‐110	Class a rhodopsin G‐protein coupled receptor gprop1	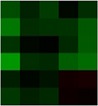
Exp1	DM06155	.00165	4.0E‐17	Class a rhodopsin G‐protein coupled receptor gprop1	
Exp1	DM06131	.00137	1.0E‐31	Class a rhodopsin G‐protein coupled receptor gprop1	
Exp1	DM12611	.00087	1.2E‐07	Class a rhodopsin G‐protein coupled receptor gprop2	
Exp2	JAN36420	.00120	1.2E‐07	Class a rhodopsin G‐protein coupled receptor gprop2	
Exp2	KZS21495	.00100	1.5E‐63	Blue wavelength opsin	

Note

All protein accessions represent significant matches (E = 10^–5^) to *Daphnia magna* protein coding sequences. Fold change is represented as log_2_ values. Heatmaps match to each target transcript. The designations “#L:#D” represent the number of hours of light and dark per day, respectively.

## 4 DISCUSSION

The experimental results provide strong evidence that differences in photoperiod alone, that is without the presence other cues (e.g., temperature, crowing, low nutrition), caused substantial changes in life history strategies, including (a) a decreasing trend in total fecundity (Figure 1a); (b) significantly increased male production (Figure 1b); and (c) indication of significantly increased metabolic rate (Figure 2) in the short‐day length (≤8‐hr daylight) photoperiods. Further, *Daphnia magna* that were acclimated to the various photoperiods displayed differences in instantaneous responses to light in phototaxis assays. Generally, *D. magna* that were acclimated to short‐day length (≤8‐hr daylight) were more averse to and avoided light (Figure 3a,c) and swam faster (Figure 3b,d) when in light conditions, where both of these responses were enhanced in younger individuals. Transcriptomic investigations identified significant differences in transcript expression in all photoperiods relative to the control 16L:8D photoperiod where expression profiles within the predominant KEGG ontology categories were the most similar among the short‐day length and equinox photoperiods (≤12‐hr daylight) exposures (Figure S10). Further, gene transcripts putatively identified to be involved in the various organism‐level responses (e.g., circadian rhythm, sex determination and egg processes) to the photoperiod exposures had unique expression profiles in the short‐day length and equinox photoperiods relative to the control (Table 1). These responses were putatively connected to observed life history phenotypes as well as phototaxis behaviours (Table 2). In the following discussion, we provide context for the various responses to photoperiod with regard to the sensing of environmental cues, life history strategy and the basic ecology of *D. magna*.

**Table 2:** Connecting observations in *Daphnia magna* to functional genomic expression to provide inferences about mechanistic responses to photoperiod

Observations in *D. magna*		Genomics (Transcriptional expression)	
Life history endpoints	Photoperiod effects	Functions	Functional response(s)
Metabolic rate	Increased in (8L:16D, 4L:20D)	Circadian rhythm	Period circadian protein (PER) and a protein coding F‐box/LRR‐repeats (possibly JET), each of which are critical in establishing Circadian clock, were affected by photoperiod (Koh et al., 2006; Zerr et al., 1990). Photoperiod influences to circadian rhythm are critical to establishing periodic activity and metabolism and likely influence the majority of responses observed herein
		Metabolism	Differential expression of various pathways involved in carbohydrate, amino acid/ protein and fatty acid metabolism which are critical in establishing system‐level energy metabolism
Growth	Decreased carapace length (4L:20D)	Cuticle and chitin (development, molting, reproduction and more)	Increased expression of cuticular protein analogous to peritrophins 1‐G (CPAP1G) in the short‐day length photoperiod exposures. Peritrophin 1 (CPAP1) family is essential for development, molting, cuticle integrity, locomotion and fecundity in arthropods (Jasrapuria et al., 2012)
Reproduction	Decreased neonates (4L:20D)	Egg	Transcriptional expression in short‐day length included: (a) Decreased expression of two vitelline membrane proteins which serve as a source chorion proteins important in early development (Pascucci et al., 1996), (b) Decreased expression of chorion peroxidase activity facilitates cross‐linking of chorionic proteins during the “hardening” phase of egg development (Mindrinos et al., 1980), (c) Increased expression of vitellogenin fused with the superoxide dismutase where vitellogenin is a critical marker of Daphnia fecundity (Ebert, 1993), and (d) Decreased transcriptional expression of the female sterile 2, and inhibitor of oogenesis (Schüpbach & Wieschaus, 1991)
		Cuticle and chitin (development, molting, reproduction and more)	Cuticular protein analogous to peritrophins 1‐G (CPAP1G) had increased expression in the short day‐length photoperiod where (CPAP1) family is essential for fecundity in arthropods (Jasrapuria et al., 2012)
Male production	Increased (12L:12D, 8L:16D and 4L:20D)	Environmental signalling	Decreased expression of glutamate receptor ionotropic, kainate. This target is an inhibitor of glutamate signaling in mammals (Contractor et al., 2000), which if this function is conserved in *D. magna*, suggests a putative increase in glutamate signaling. Glutamate signaling induces male production in the *Daphnia pulex* WTN6 strain (Toyota, Miyakawa, Hiruta, et al., 2015) and is a photoperiod‐sensitive mechanism (Toyota, Miyakawa, Hiruta, et al., 2015)
		Sex determination	Decreased transcriptional expression for the transformer‐2 sex‐determining protein in the short‐day photoperiod. Differential exon splicing for transformer in *Drosophila melanogaster* leads to female‐specific protein products in females and not males (Lopez, 1998). Involvement of transformer in *D. magna* sex determination is inconclusive
		Genetic information processing	Decreased expression for multiple small nuclear ribonucleoprotein particles (snRNPs) and non‐snNRP splicing factors in short day exposures. Arthropod sex determination has also been observed to be controlled by alternative splicing, although this mechanism remains to be tested in *D. magna* for sex determination genes including transformer (dmagtra, Kato et al., 2010)
**Behavior**	**Photoperiod effects**	**Functions**	**Functional response(s)**
Phototaxis experiments ‐ light avoidance (duration)	7 days old Daphnia ‐ Increased, all but 8L:16D	Visual cues	Decreased expression of blue wavelength opsin (KZS21495) and class a rhodopsin G‐protein coupled receptor 2, which are not typically involved in circadian rhythms (Zerr et al., 1990) but may be involved in short‐term light responses
	21 days old Daphnia ‐ Increased at 4L:20D and 20L:4D	Circadian rhythm	Period circadian protein (PER) and a protein coding F‐box/LRR‐repeats (possibly JET), each of which are critical in establishing Circadian clock, were affected by photoperiod (Koh et al., 2006; Zerr et al., 1990)
Phototaxis experiments ‐ light avoidance (swim velocity)	7 days old Daphnia ‐ Increased, all but 20L:4D	Environmental signalling	Decreased expression of glutamate receptor ionotropic, kainate (an inhibitor or glutamate signaling) in response to short‐day photoperiods. In Drosophila, balanced glutamate signaling dynamics among lateral and dorsal clock neurons mediate light‐avoidance behaviours and establish entrainment of light‐responsive circadian rhythms (Collins et al., 2012)
	21 days old Daphnia ‐ Increased at 4L:20D and 12L:12D	Genetic information processing	Decreased expression of nearly every transcript associated with the spliceosome. Alternative mRNA splicing of the Down syndrome cell adhesion molecule (Dscam) homolog in *Daphnia* can affect in nervous system structure/function (Brites et al., 2008)

### 4.1 General impacts of photoperiod

#### 4.1.1 Photoperiod effects on life history traits

Photoperiod had no statistically significant effects on *D. magna* survival and caused only a minor, yet statistically significant, decreasing trend in fecundity with decreasing day length (Figure 1) over the course 21‐day experiments. Little impact on basic survival from a relatively minor stressor (but important environmental cue) such as photoperiod is supported in previous studies (Korpelainen, 1986). The trend of a decreased number of total neonates produced in short‐day photoperiods observed in the present study (Figure 1a) is also supported by the literature for *Daphnia* species (Parker, 1966). The most dramatic effect of photoperiod on life history traits was the interruption of cyclic parthenogenesis resulting in significantly greater percentage of male offspring produced (Figure 1b) in short‐day length and equinox photoperiods (4L:20D, 8L:20D, 12L:12D). Korpelainen (1986) also observed that photoperiod affected male production in *D. magna* where, converse to our observations, their results showed increased male production at the 16L:8D photoperiod relative to a short‐day length photoperiod (10L:14D). It should be noted that this observation occurred predominantly at low water temperatures (14 and 19°C) relative to those used herein (25°C). In the present study, decreased reproductive output may have been influenced by increased male production in the shortest day length photoperiod where Ginjupalli and Baldwin (2013) observed that *D. magna* exposed to a hormone analog that induced male production also decreased overall fecundity. Previous investigations combining temperature and photoperiod stimuli concluded temperature was the driving factor in affecting life history traits, such as survival, reproduction, growth brood times and male production (Armitage & Landau, 1982). The present study demonstrates that photoperiod entrainment alone can affect life history strategy in *D. magna* through the course of a 21‐day exposure.

#### 4.1.2 Metabolic rate

To our knowledge, this is the first study to investigate the effect of photoperiod alone on an indicator of *Daphnia* metabolic/ bioenergetics rate. Based on literature searches, previous work integrated temperature and photoperiod as a combinational cue. Interestingly, our results indicate slight, yet statistically significant, increases in sugar metabolism as an indicator of overall metabolic rate in the two short‐day length exposures (Figure 2) when ex situ measurements were taken during entrained daylight hours. We hypothesize that this is related to a circadian‐like response where behavioural urgency might be increased in short‐day length photoperiods (indicative of autumn/ senescence seasonal cues), though additional data generation is needed to test this hypothesis. In concert with this hypothesis, these short‐day length photoperiod treatments produced more male progeny, which may be more energetically expensive to produce, necessitating increased metabolic rates. Again, this needs to be tested in dedicated experiments.

#### 4.1.3 Photoperiod effects on phototaxis

It is well documented that *Daphnia* species are generally photonegative (Dodson et al., 1997; Van Gool & Ringelberg, 1995), although there are some reports of photopositive clones (Michels et al., 1999; Ringelberg, 1964). Prior to this study, little was known about the effect of long‐term (7–21 days) entrainment to various photoperiods on *D. magna* phototactic swimming velocity and duration of times spent in different light intensities. Photoperiod significantly affected phototaxis responses in *D. magna* where the photonegative responses were most sensitive in younger (7 days old) versus older (21 days old) *D. magna* (Figure 3). Meester (1992) similarly observed differential phototaxis where juveniles tended to be more photonegative than adults, even in clones exhibiting photopositive responses as adults. According to previous studies (e.g., Van Gool & Ringelberg, 1995), negative phototaxis responses lead to typical *Daphnia* diurnal water‐column migration patterns which are hypothesized to optimize predatory avoidance during light conditions. The 7‐day‐old *D. magna* in the present study exposed to all photoperiods except for the extreme long‐day length (20L:4D) swam significantly faster when in light conditions (approximately 7–9 mm/s), relative to dark conditions (approximately 5–6 mm/s; Figure 3). Faster swimming in the light is supported in the literature by Dodson et al. (1997) and is presumably a direct negative phototaxis response to avoid predators in light conditions and/or speed the process of moving out of the light. Overall, photoperiod was shown to affect phototaxis in *D. magna* with the most pronounced photonegative responses occurring at extreme photoperiods and in young life stages.

### 4.2 Transcriptomic response to photoperiod on generalized functions

Kyoto Encyclopedia for Genes and Genomes orthology annotations provided insights into generalized functions affected by photoperiod (Figure S9). Below we discuss observations from the three major KO classes: environmental signalling, genetic information processing and metabolism.

#### 4.2.1 Environmental signalling

**Figure 4:**
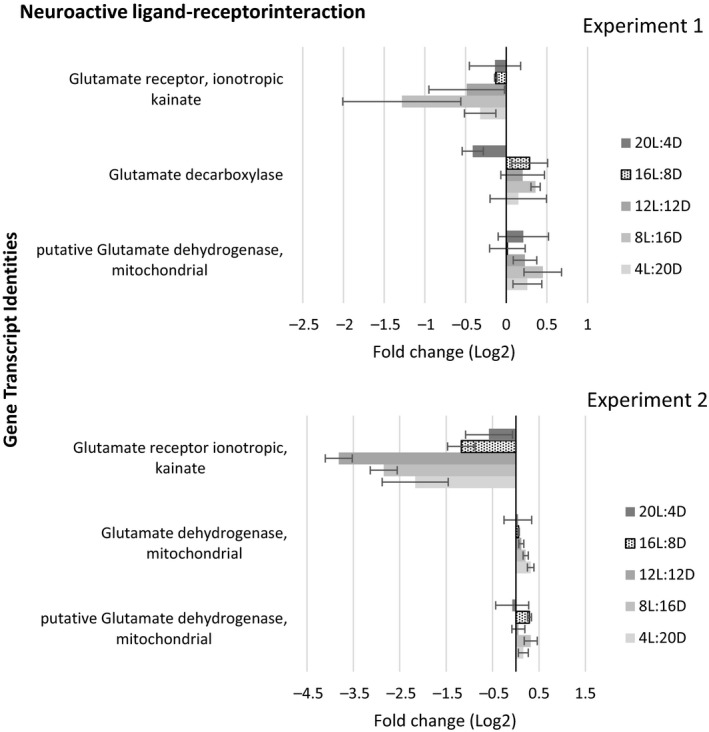
Transcript expression for gene targets having significant differential expression in response to photoperiod relative to the 16L:8D control that were involved in neuroactive ligand–receptor interactions (NLRI). Association with NLRI is based on KEGG orthology (KO) annotations. Fold changes represent means of all replicates, and error bars represent one standard deviation. Results provided for each individual experimental block

The pathways included within the KEGG environmental information processing ontology (https://www.genome.jp/kegg/pathway.html#environmental) represent various first responders to critical environmental stimuli. Within this ontology, “signalling molecules and interactions” represents the rapid‐response elements capable of instantaneous reaction to dynamic environmental cues, including light stimuli. Thus, photoperiod‐induced transcriptional expression changes within this signalling molecule cascade, including neuroactive ligand receptors (Figure S10), were not surprising. The neuroactive ligand–receptor pathway affects neural signalling facilitating the most rapid whole‐organism responses in biology where decreased transcriptional expression of glutamate receptor ionotropic, kainate was observed in response to shortened day photoperiods (Figure 4). Glutamate receptor ionotropic, kainite is an inhibitor of glutamate signalling in mammals (Contractor, Swanson, Sailer, O'Gorman, & Heinemann, 2000); thus, if this function is orthologous in *D. magna*, the decreased transcriptional expression in short‐day photoperiods suggests the potential for increased glutamate signalling. In *Drosophila*, balanced glutamate signalling dynamics among lateral and dorsal clock neurons mediate light‐avoidance behaviours and establish entrainment of light‐responsive circadian rhythms (Collins, Kane, Reeves, Akabas, & Blau, 2012). A broad suite of glutamate response elements have been identified in *Daphnia pulex;* thus, glutamate signalling may similarly play a role in light‐avoidance and circadian behaviours in *Daphnia* species (Miyakawa, Sato, Colbourne, & Iguchi, 2015). Additionally, recent findings have demonstrated glutamate signalling induces male production in the *D. pulex* WTN6 strain, but not other *D. pulex* strains (Toyota, Miyakawa, Yamaguchi, et al., 2015), a response comparatively attributable to methyl farnesoate (putative juvenile hormone in daphnids) exposure (Toyota, Miyakawa, Hiruta, et al., 2015). Given the transcriptional evidence for potential increased glutamate signalling and the increased male production observed in the *D. magna* strain tested in present study, similarity in photosensitive mechanisms of male production is plausible between *D. magna* and the *D. pulex* WNT6 strain. If fact, a recently published study by Camp and Haeba (2019) indicated that photoperiod‐dependent methyl farnesoate status indeed provided permissive conditions for male production in *D. magna*, but the ultimate regulatory trigger was temperature where male production increased with temperature in *D. magna* and the opposite was true in *D. pulex*.

#### 4.2.2 Genetic information processing

The short‐day length and equinox photoperiod exposures caused predominantly decreased expression for gene transcripts involved in multiple facets of genetic information processing (Figure S10). A striking observation was the decreased expression of nearly every transcript associated with the spliceosome (Figure S11). Spliceosomes are large complex ribonucleoproteins (RNPs) present in eukaryotes that facilitate splicing of introns from pre‐mRNA as well as alternative splicing of exons to express multiple forms of an mRNA from a single gene (Will & Luhrmann, 2011). The transcriptomics for *D. magna* indicated decreased expression, predominantly in the short‐day and equinox photoperiods, for multiple small nuclear ribonucleoprotein particles (snRNPs) and non‐snNRP splicing factors (Figure S11) that make up the spliceosome indicating potentially reduced capacity for spliceosome‐based pre‐mRNA processing (Black, Chabot, & Steitz, 1985; Blencowe, Bowman, McCracken, & Rosonina, 1999).

In the human genome, alternative exon splicing has been identified to be a major contributor to proteome diversity where estimates range from 40% to 80% of genes having alternative splicing potential (Matlin, Clark, & Smith, 2005). Extensive alternative exon splicing has been observed in *Daphnia* for specific genes, such as the Down syndrome cell adhesion molecule (Dscam) homolog, where 13,000 unique transcripts are formed (Brites et al., 2008). Similar to observations in insects, the alternative exon splicing of Dscam in *Daphnia* has been implicated in playing a critical role both in immune responses and in nervous system structure/ function (Brites et al., 2008). In certain species, arthropod sex determination has also been observed to be controlled by alternative splicing, although this mechanism remains to be tested in *D. magna* for sex determination genes including transformer (dmagtra, Kato et al., 2010). A possible splice variant of a transformer homolog was recently identified in *Daphnia galeata* where expression showed female bias (Huylmans, Lopez Ezquerra, Parsch, & Cordellier, 2016), providing additional observations of potential gene splicing‐based sex determination mechanisms in the *Daphnia* genus. The specific effects of short‐day length photoperiods on spliceosome activity in *D. magna* and resultant phenotypes require dedicated testing. Presently, possible outcomes of the overall decreased expression include overall differential exon splicing which may affect sex‐specific phenotypes, determination of neural state/ function and potential effects on immunity.

#### 4.2.3 Metabolism

Gene transcripts involved in a diverse suite of metabolic pathways were differentially expressed in response to different photoperiods, including effects on various carbohydrates, amino acids/ proteins, nucleic acids, fatty acids and etcetera (Figure S10). Photoperiod proved to be an influential environmental stimulus affecting reproductive output, male production, energy expenditure and phototaxis (Figures 1, 2, 3, 4). Such responses are underpinned by diverse suborganismal metabolic responses orchestrating the allocation of resources and energy towards the overall suite of life history traits (Ananthasubramaniam et al., 2015). Similar to the results of the present study, Toyota et al. (2016) demonstrated that the short‐day length photoperiod caused differential expression of gene transcripts within various KEGG metabolomic pathways including alanine, aspartate and glutamate metabolism, starch and sucrose metabolism, amino sugar and nucleotide sugar metabolism, and galactose metabolism. Many of the common responses between our study and the Toyota et al. (2016) investigation were related to system‐level energy metabolism. Effects of equinox and/or short‐day photoperiod on circadian rhythms (i.e., increased phototaxis; Figure 3) and life history strategies (i.e., increased male production; Figure 1) undoubtedly influenced energy utilization in *D. magna* as evidenced by increased energy expenditure in the short‐day length photoperiods (Figure 2). Overall, the transcriptional evidence suggests changes in multiple metabolic pathways that putatively drive the functional molecular mechanisms underlying the organismal response in *D. magna* that are described above, systemically initiated through environmental sensing/signalling and subsequent genetic information processing.

### 4.3 Finding functions – connecting omics to organism‐level responses

Similar to the use of KEGG orthology annotations, we leveraged the annotation text mining results to connect gene functions to observed organism‐level responses (Table 2). Below we discuss observations related to circadian rhythm; sex determination; cuticle/chitin processes related to development, moulting, reproduction; egg production; and visual cues.

#### 4.3.1 Circadian rhythm

The decreased expression of period circadian protein (PER), which is involved in controlling circadian rhythms in *Drosophila* (Zerr, Hall, Rosbash, & Siwicki, 1990), was observed in short‐day length photoperiods (Tables 1 and 2) providing evidence of potential processes underlying circadian cycling in *D. magna*. The cyclic expression of PER in the photoreceptor nuclei, glial cells and neurons of *Drosophila* has been implicated in driving circadian rhythms of diurnal activity and is recognized to be influenced by light/dark cycles (Zerr et al., 1990). Curtin, Huang, and Rosbash (1995) demonstrated that this cyclic expression of PER in *Drosophila* was mediated through a negative feedback loop where PER protein inhibits transcriptional expression of PER mRNA in a time‐series dynamic cycle. In the present study, the *Daphnia* RNA samples were collected within an hour after initiation of the light phase of each photoperiod; thus, the observed PER transcript expression represents a measurement early within the potential PER transcript/protein feedback cycle. If analogous processes observed in *Drosophila* were operating in *D. magna*, PER protein likely accumulated during the extended dark cycle of the short‐day exposures, thus increasing the stock of PER protein in tissues resulting in inhibited PER transcript expression. Additionally, an F‐box protein with leucine‐rich repeats (F‐box/LRR‐repeat) termed JET has been identified in *Drosophila* to confer light sensitivity of the circadian clock (Koh, Zheng, & Sehgal, 2006). In our study, *D. magna* had significant changes in expression for two unique transcripts coding F‐box/LRR repeats (KZS07878, DM12109) where expression was either increased or decreased in the short‐day photoperiod exposures, depending on the experimental trial (Table 1). If the function is homologous to *Drosophila*, differential expression of F‐box/LRR repeats potentially reflects light‐responsive circadian clock changes.

#### 4.3.2 Sex determination

As described in the Environmental Sensing and Genetic Information Processing sections above, glutamate signalling pathways and alternative mRNA transcript splicing mechanisms, respectively, potentially contribute to the male production observed in the present experiment. Additionally, decreased transcriptional expression for the transformer‐2 sex‐determining protein (JAN91947; Table 1) was observed in concordance with increased male production (Figure 1) in *D. magna* exposed to short‐day length photoperiods. Differential exon splicing of the transformer (tra) and Sex‐lethal (Sxl) genes in *Drosophila melanogaster* led to production of female‐specific protein products in females, but not males (Kato et al., 2010). Kato, Kobayashi, Watanabe, and Iguchi (2011) conducted a genomic study of the transformer (tra) gene in *D. magna* (dmagtra) which indicated no differential expression or exon splicing among males and females, although a potential splice variant of tra was observed in *Daphnia galeata* that did show some degree of female bias (Huylmans et al., 2016). Finally, the doublesex (Dsx) gene (DapmaDsx1) has been observed to control *D. magna* sex determination, where increased expression was associated with male formation (Kato et al., 2011). In the present study, Dsx probes were included in the microarray, but were not differentially expressed in response to the short‐day length photoperiods.

#### 4.3.3 Cuticle and chitin (development, moulting, reproduction and more)

Chitin is an extracellular polysaccharide matrix that represents the predominant structural component of arthropod exoskeletons (Tetreau et al., 2015). The direction and magnitude of expression for multiple components of chitin metabolism and cuticle structure (chitin plus proteins) in *D. magna* varied in response to photoperiod (Table 1). The expression of the cuticular protein analogous to peritrophins 1‐G (CPAP1G) tended to have increased expression in the short‐day length photoperiod exposures (Table 1). RNAi investigations in the cuticular protein analogous to peritrophin 1 (CPAP1) family of proteins in the beetle *Tribolium castaneum* indicated that several members were essential for insect development, moulting, cuticle integrity, locomotion or fecundity (Jasrapuria, Specht, Kramer, Beeman, & Muthukrishnan, 2012). Given that cuticle remodelling is dynamic in arthropods to facilitate periodic moulting cycles enabling growth, the dynamic expression of the various chitin metabolism enzymes and cuticle proteins in *D. magna* is intuitive given the life history changes occurring across photoperiods.

#### 4.3.4 Egg

Given that neonate production was decreased by roughly 12% in the most extreme short‐day length photoperiod, we investigated transcriptional expression relevant in egg productions to provide potential mechanistic insights. Transcript expression for genes coding vitelline membrane proteins (JAN90603, KZS10607) was decreased in the short‐day length photoperiods (Table 1). The vitelline membrane has been demonstrated to act as the reservoir for chorion proteins during early developmental stages of *Drosophila* egg development (Pascucci, Perrino, Mahowald, & Waring, 1996). Chorion peroxidase precursors (JAN29198, JAN477987) also had decreased expression in short‐day length photoperiods (Table 1). Mindrinos, Petri, Galanopoulos, Lombard, and Margaritis (1980) demonstrated that chorion peroxidase activity facilitates cross‐linking of chorionic proteins during the “hardening” phase of egg development in *Drosophila*. In contrast to the decreased expression of transcripts related to the egg “shell,” the expression of vitellogenin fused with the superoxide dismutase (BAE94324) was increased in the short‐day length photoperiods (Table 1). This gene complex (vitellogenin fused to the superoxide dismutase‐like domain) in *D. magna* represents a major component of yolk proteins in eggs (Kato, Tokishita, Ohta, & Yamagata, 2004) where vitellogenesis is recognized as a critical determinant of *Daphnia* egg production rate and quality (Ebert, 1993). Additionally, decreased transcriptional expression of the female sterile 2 (KZS06734) gene was observed in *D. magna* in the short‐day length photoperiods (Table 1). Female sterile mutations in *Drosophila* have been observed to impair reproduction by inhibiting egg production by blocking oogenesis or by impairing egg morphology (Schüpbach & Wieschaus, 1991). Finally, notch (DM03056) expression was increased in short‐day length photoperiod exposures (Table 1), where notch is critical in *Daphnia* segmentation and overall organismal development (Eriksson, Ungerer, & Stollewerk, 2013). Overall, a variety of functions critical in egg production and development were differentially expressed at the transcriptional level in response to short‐day length photoperiod. Given changes in energy expenditure (Figure 2), male production (Figure 1) and overall neonate production (Figure 1) in short‐day length and equinox photoperiods, shifts in the expression of genes underlying reproductive physiology provide insights into processes influenced/affected by photoperiod.

#### 4.3.5 Visual cues

There were significant, albeit low relative decreases in transcriptional expression of blue wavelength opsin (KZS21495) and class a rhodopsin G‐protein coupled receptor 2 (JAN36420) in the short‐day length photoperiods. Rhodopsin dynamics in *Anopheles gambiae* mosquitoes were observed in response to light/dark exposures, where the “mature” rhodopsin protein tended to increase in concentration within the major R1‐6 photoreceptor class of the mosquito eye in response to dark exposure (Moon, Metoxen, Leming, Whaley, & O'Tousa, 2014). In the present study, it is plausible that dynamics in rhodopsin accumulation in light/dark cycles could have implications related to phototaxis, including the observation of intensified light aversion (Figure 3).

## 5 CONCLUSIONS

In *Daphnia magna*, photoperiod as a sole environmental cue had powerful influence over phototaxis behaviours and multiple life history outcomes where functional genomic analysis identified a variety of logical connections to potential underlying molecular mechanisms (a synthesis of connections is provided in Table 2). Entrainment to short‐day photoperiods caused *D. magna* to have significantly increased light‐avoidance behaviours relative to conspecifics reared in the control (long‐day photoperiod – 16L:8D) where young *Daphnia* (7 days old) displayed the most pronounced light‐avoidance responses. Functional transcriptomic analysis identified photoperiod‐induced differential expression of gene targets involved in glutamate signalling, which is critical in arthropod light‐avoidance responses, as well as period circadian protein and proteins coding F‐box/LRR‐repeat domains, all of which contribute to establishing circadian rhythms. Short‐day photoperiods also produced indications of increased metabolic rate during light hours which corresponded with broadscale changes in expression across multiple pathways that establish system‐level energy metabolism. A striking life history response to photoperiod was increased male production across short‐day and equinox photoperiods (4L:20D, 8L:16D and 12L:12D). Correspondingly, transcriptional expression consistent with multiple putative mechanisms of male production was observed including photoperiod‐dependent transcriptional expression of transformer‐2 sex‐determining protein (Lopez, 1998) and associated small nuclear ribonucleoprotein particles (snRNPs) involved in splice variant expression for genes such as transformer. Additionally, changes in transcriptional expression that were suggestive of increased glutamate signalling show parallelism to responses observed to induce male production in *Daphnia pulex* (Toyota, Miyakawa, Yamaguchi, et al., 2015) via photoperiod‐sensitive mechanisms (Camp & Haeba 2019; Toyota, Miyakawa, Hiruta, et al., 2015). Overall, the results demonstrate the importance of photoperiod on behaviour and life history trajectories in *D. magna* where we have now established multiple putative mechanistic pathways underlying these critical responses.

## CONFLICT OF INTEREST

The authors declare no conflict of interest or relationship, financial or otherwise that might be perceived as influencing the authors' objectivity in the reporting of the study results or interpretations.

## AUTHOR CONTRIBUTIONS

The study idea was conceived by K.G., A.K., M.K. and T.S. The overall project was managed by K.G. The study equipment was constructed by K.G., A.K. and M.W. All *Daphnia* exposures were managed and the life history and behaviour results were interpreted by A.K. The genomic analyses were conducted and the genomics and overall results were interpreted by K.G. The *Daphnia* exposures were conducted by J.L. *Daphnia* samples were collected, RNA was extracted and quality assessed RNA was performed by M.W. and M.B. Microarray hybridizations were conducted and data from microarrays were extracted by X.G. The metabolic rate assays were conducted by N.M. Reannotation of *Daphnia magna* microarray was performed by L.B. The manuscript was developed by K.G., A.K., M.K. and T.S.

## Data Availability

All microarray data/results are publicly available at the Gene Expression Omnibus (GEO, http://www.ncbi.nlm.nih.gov/geo/info/linking.html) under series accession GSE117244.
