# Supplementary material for: Different as night and day: Behavioural and life history responses to varied photoperiods in Daphnia magna
Source: Mol Ecol. 2019 Sep 26;28(19):4422–38. doi: 10.1111/mec.15230 (PMC6856852; doi:10.1111/mec.15230)
Supplement: Supplementary file 1 [file MEC-28-4422-s001.docx]

***Supplemental Figures***

***Title:* Different as Night and Day: Behavioral and Life History Responses to Varied Photoperiods in *Daphnia magna***

***Running Title:*** Mechanistic Responses to Photoperiod

***Authors:***

Kurt A. Gust^1A*^, [Kurt.A.Gust@usace.army.mil](mailto:Kurt.A.Gust@usace.army.mil)

Alan J. Kennedy^1A^, [Alan.J.Kennedy@usace.army.mil](mailto:Alan.J.Kennedy@usace.army.mil)

Jennifer G. Laird^1^, [Jennifer.G.Laird@usace.army.mil](mailto:Jennifer.G.Laird@usace.army.mil)

Mitchell S. Wilbanks^1^, [Mitchell.S.Wilbanks@usace.army.mil](mailto:Mitchell.S.Wilbanks@usace.army.mil)

Natalie D. Barker^2^, [Natalie.D.Barker@usace.army.mil](mailto:Natalie.D.Barker@usace.army.mil)

Xin Guan^2^, [Xin.Guan@usace.army.mil](mailto:Xin.Guan@usace.army.mil)

Nicolas L. Melby^1^, [Nicolas.L.Melby@usace.army.mil](mailto:Nicolas.L.Melby@usace.army.mil)

Lyle D. Burgoon^1^, [Lyle.D.Burgoon@usace.army.mil](mailto:Lyle.D.Burgoon@usace.army.mil)

Michael E. Kjelland^1,3,4^, [michael.kjelland@mayvillestate.edu](mailto:michael.kjelland@mayvillestate.edu)

Todd M. Swannack^1^, [Todd.M.Swannack@usace.army.mil](mailto:Todd.M.Swannack@usace.army.mil)

***Author Affiliations:***

^1^ US Army, Engineer Research and Development Center, Environmental Laboratory, Vicksburg, MS, 39180, USA.

^2^ Bennett Aerospace, Cary, NC, 27511, USA.

^3^ Current Affiliation: Mayville State University, Division of Science & Mathematics, Mayville, ND, 58257, USA

^4^ Current Affiliation: Conservation, Genetics & Biotech, LLC, Valley City, ND, 58072, USA

^A^ Gust and Kennedy should be considered joint first authors.

*Corresponding Author

Phone: 601-634-3593

E-mail: [kurt.a.gust@usace.army.mil](mailto:kurt.a.gust@usace.army.mil)

Figure S1. Experimental chambers were custom fabricated for this project. The rectangular chambers were able to accommodate 1L of exposure media and three chambers were housed in each photoperiod box during experiments. The photoperiod boxes were sealed to administer each experimental photoperiod using light cycle timers. A high resolution camera was used to assess *Daphnia* position in the photoperiod boxes in a time series lasting 1.5 hours where position was assessed relative to the bottom of the exposure chambers. Real-time investigation of light versus dark preference was conducted by placing individual Daphnia in custom-fabricated swim chambers which were tracked using Ethovision software.


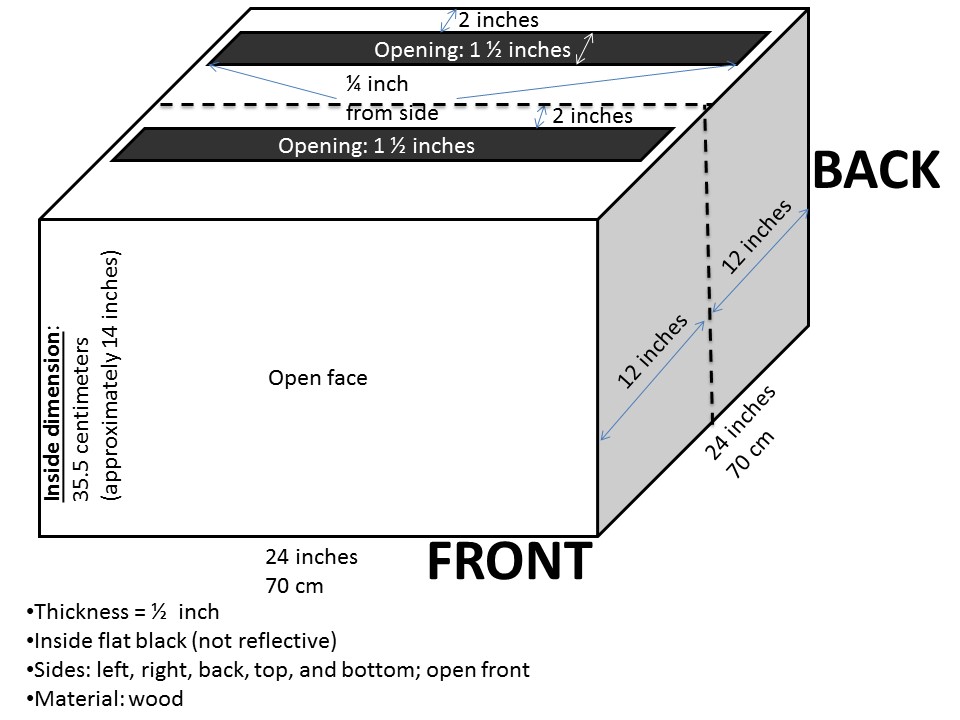


Figure S2. Diagram of photoperiod exposure boxes.


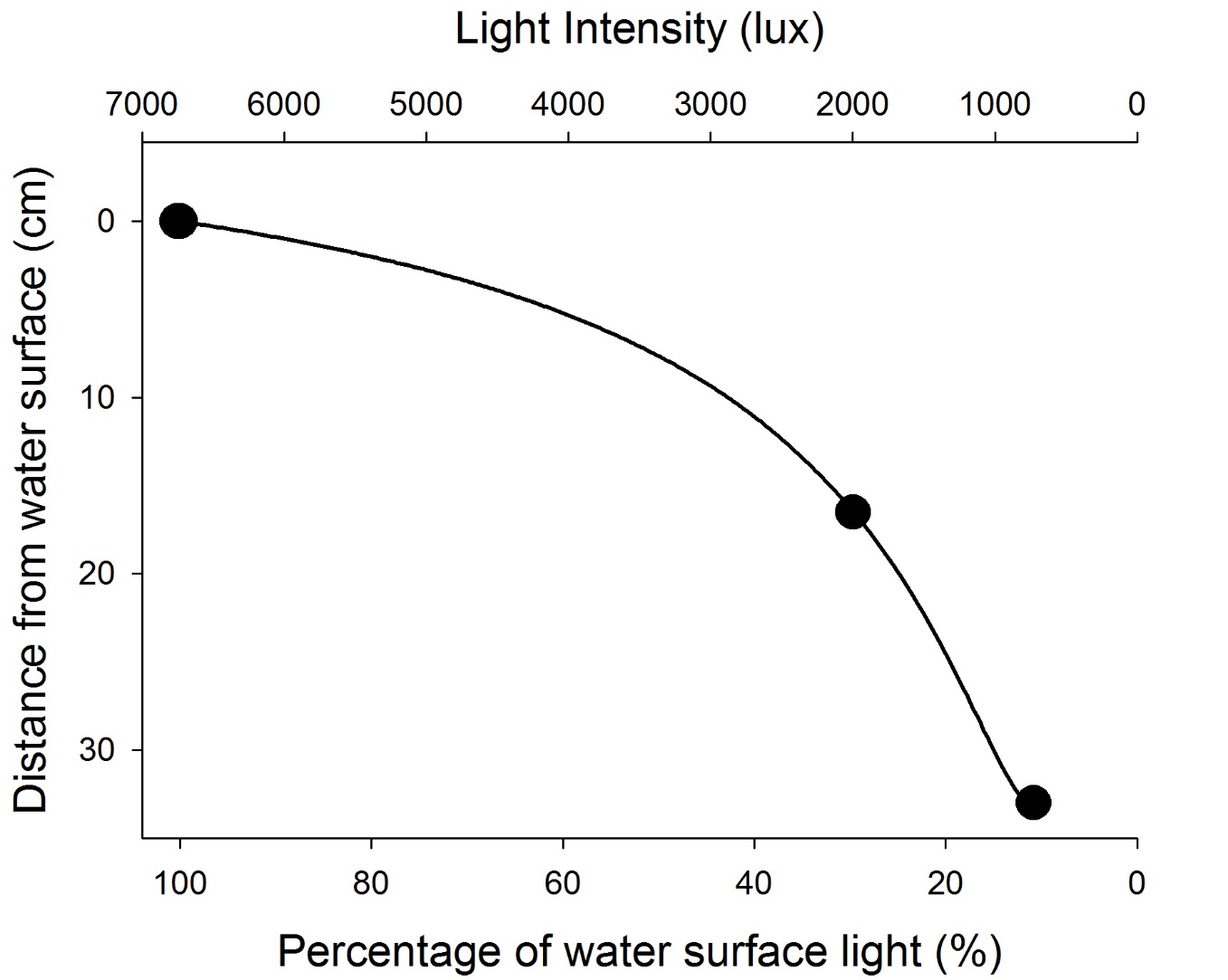


Figure S3. Penetration of fluorescent light into test chambers.


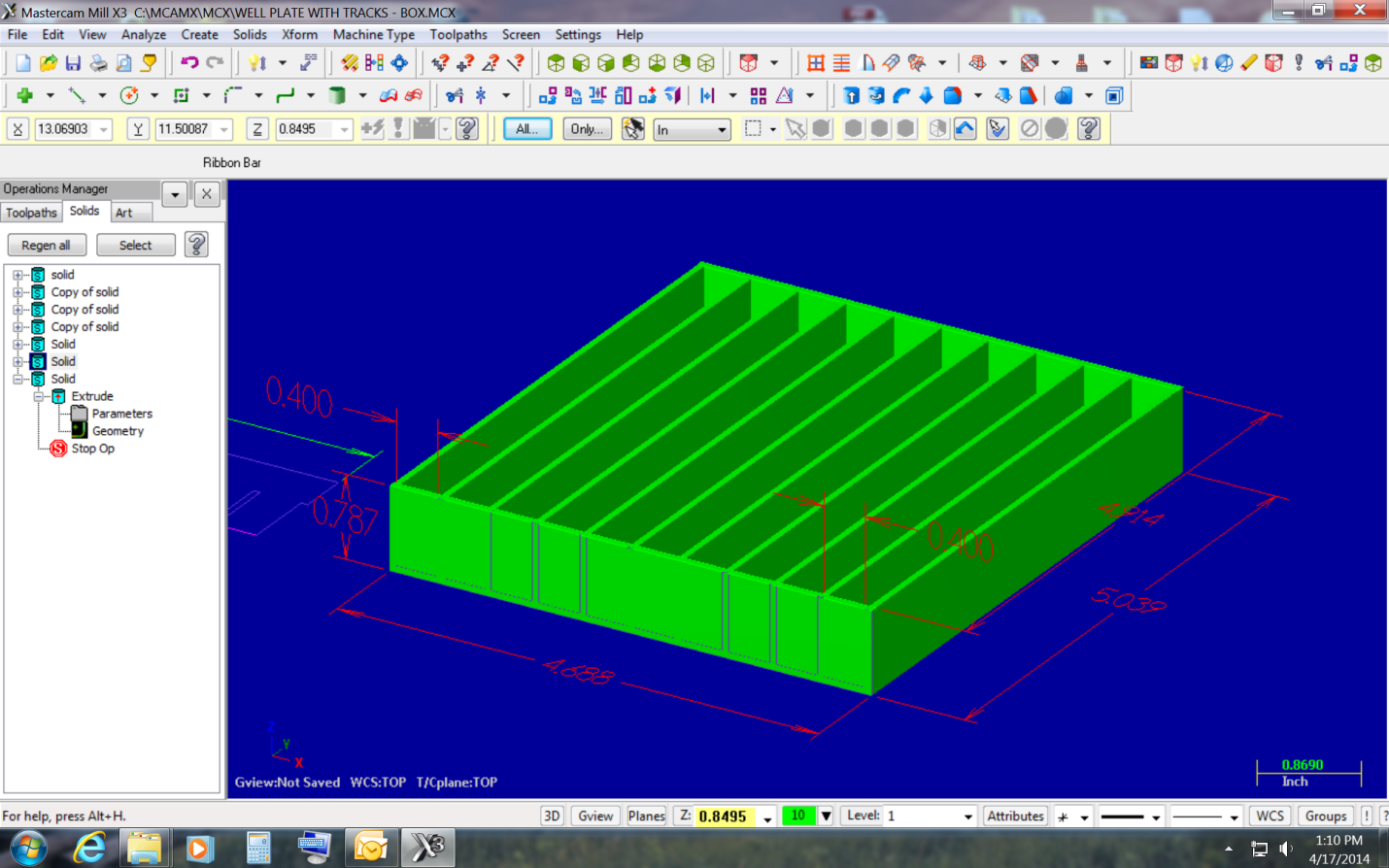


Figure S4. Diagram of phototaxis chamber.

Figure S5. *Daphnia magna* carapace length in the different photoperiod treatments. Asterisks denote a statistically significant (p = 0.05) different relative to the control (16L:8D).

A B

**Experiment 1 – 21 day old *Daphnia***

**Experiment 1 – 7 day old *Daphnia***

C D

**Experiment 2 – 21 day old *Daphnia***

**Experiment 2 – 7 day old *Daphnia***

Figure S6. Phototaxis investigation providing time spent in light versus dark zones in the phototaxis chamber for experimental trials 1 and 2 (each included n = 3) for (A) 7 day old and (B) 21 day old *D. magna*. Black bars indicate the dark zone while white bars indicate the light zone. The “S” and “NS” designations denote significant and non-significant differences (p = 0.05), respectively, in time spent in light versus dark zones within each photoperiod.

A B

**Experiment 1 – 21 day old *Daphnia***

**Experiment 1 – 7 day old *Daphnia***

C D

**Experiment 2 – 21 day old *Daphnia***

**Experiment 2 – 7 day old *Daphnia***

Figure S7. Phototaxis investigation providing swim velocity in light versus dark zones in the phototaxis chamber for experimental trials 1 and 2 (each included n = 3) for (A) 7 day old and (B) 21 day old *D. magna*. Black bars indicate the dark zone while white bars indicate the light zone. The “S” and “NS” designations denote significant and non-significant differences (p = 0.05), respectively, in swim velocity in light versus dark zones within each photoperiod.

Figure S8. Principle component analysis of transcript expression results.

Figure S9. KEGG ontology (KO) annotations for experimental photoperiods. Pie charts provide proportion of significant differentially expressed transcripts (p = 0.01) contributing to each primary KO.

Figure S10. Transcript expression and hierarchical clustering of transcripts within the three major KEGG ontology categories. Labels display KEGG pathway associations.

Supplemental Figure S11. Transcript expression for gene targets having significant differential expression in response to photoperiod relative to the 16L:8D control that were involved in spliceosome processes. Association with the spliceosome is based on KEGG orthology (KO) annotations. Fold changes represent means of all replicates and error bars represent one standard deviation.
